# Examination of clinical and environmental *Vibrio parahaemolyticus* isolates by multi-locus sequence typing (MLST) and multiple-locus variable-number tandem-repeat analysis (MLVA)

**DOI:** 10.3389/fmicb.2015.00564

**Published:** 2015-06-10

**Authors:** Catharina H. M. Lüdeke, Narjol Gonzalez-Escalona, Markus Fischer, Jessica L. Jones

**Affiliations:** ^1^Gulf Coast Seafood Laboratory, Division of Seafood Science and Technology, Food and Drug AdministrationDauphin Island, AL, USA; ^2^Hamburg School of Food Science, University of HamburgHamburg, Germany; ^3^Division of Microbiology, Center for Food Safety and Applied Nutrition, Food and Drug AdministrationCollege Park, MD, USA

**Keywords:** *Vibrio parahaemolyticus*, phylogenetics, MLVA, MLST, HRM

## Abstract

*Vibrio parahaemolyticus* is a leading cause of seafood-borne infections in the US. This organism has a high genetic diversity that complicates identification of strain relatedness and epidemiological investigations. However, sequence-based analysis methods are promising tools for these identifications. In this study, Multi-Locus Sequence Typing (MLST) and Multiple-Locus Variable-Number Tandem-Repeat Analysis (MLVA) was performed on 58 *V. parahaemolyticus* isolates (28 of oyster and 30 of clinical origin), to identify differences in phylogeny. The results obtained by both methods were compared to Pulsed-Field Gel Electrophoresis (PFGE) patterns determined in a previous study. Forty-one unique sequence types (STs) were identified by MLST among the 58 isolates. Almost half of the isolates (22) belonged to a new ST and added to the MLST database. A ST could not be generated for 5 (8.6%) isolates, primarily due to an untypable *recA* locus. Analysis with eBURST did not identify any clonal complex among the strains analyzed and revealed 37 singeltons with 4 of them forming 2 groups (1 of them SLV, and the other a DLV). An established MLVA assay, targeting 12 total genes through three separate 4-plex PCRs, was successfully adapted to high resolution melt (HRM) analysis with faster and easier experimental setup; resulting in 58 unique melt curve patterns. HRM-MLVA was capable of differentiating isolates within the same PFGE cluster and having the same ST. Conclusively, combining the three methods PFGE, MLST, and HRM-MLVA, for the phylogenetic analysis of *V. parahaemolyticus* resulted in a high resolution subtyping scheme for *V. parahaemolyticus*. This scheme will be useful as a phylogenetic research tool and as an improved method for outbreak investigations for *V. parahaemolyticus*.

## Introduction

*Vibrio parahaemolyticus* can cause acute gastroenteritis associated with consumption of raw or undercooked seafood (Nishibuchi and DePaola, 2005). Presently, this bacterium represents the most common cause for seafood-associated infections in the United States (Iwamoto et al., 2010). *V. parahaemolyticus* has shown a high rate of recombination and mutation which leads to a high genetic diversity (Gonzalez-Escalona et al., 2008). *V. parahaemolyticus* isolates are frequently characterized for their virulence gene profile, serotype, ribotype, and/or Pulsed-Field Gel Electrophoresis (PFGE) pattern for research studies and epidemiological investigations (Broberg et al., 2011; Jones et al., 2012; Paranjpye et al., 2012; Banerjee et al., 2014; Xu et al., 2015). However, there are several phylogenetic and evolutionary methodologies for differentiation of *V. parahaemolyticus*, such as Multi-Locus Sequence Typing (MLST) and Multiple-Locus Variable-Number Tandem-Repeat Analysis (MLVA), which may provide greater discrimination.

For this study, we selected two sequence-based typing methods, MLST and MLVA. MLST is based on sequence diversity of loci which are generally well-conserved, or under purifying selection, as is the case with housekeeping genes. MLST is a frequently used typing method for many organisms and the development of a public database (PubMLST) has simplified sequence analysis and identification of evolutionary relationships within bacterial species (Maiden et al., 1998; Perez-Losada et al., 2013). MLST was selected to examine the current strain collection based on the previous success of the technique for characterizing diverse environmental and clinical *V. parahaemolyticus* isolates (Gonzalez-Escalona et al., 2008) and to add a diverse set of isolates to the MLST database. MLVA has also been used to distinguish isolates with little genetic variation. MLVA uses PCR for amplification of size polymorphisms in several Variable-Number Tandem-Repeat (VNTR) loci (Lindstedt, 2005). The VNTRs are highly polymorphic and are well suited for differentiation of bacterial isolates (Lindstedt, 2005; van Belkum, 2007). MLVA was selected for use in this study as it is able to differentiate between indistinguishable PFGE patterns for *V. parahaemolyticus* (Hayat et al., 1993; Harth-Chu et al., 2009) or identical MLST sequence types (STs) in other organisms (Maiden et al., 1998, 2013). Recently, MLVA was used for epidemiological analysis for discrimination of clinical and environmental *V. parahaemolyticus* isolates with indistinguishable Direct Genome Restriction Enzyme Analysis (DGREA) patterns (Harth-Chu et al., 2009; Garcia et al., 2012). Generally, the PCR products from MLVA are separated by agarose gel or capillary electrophoresis (CE) (Lindstedt et al., 2013). However, differentiation of amplification products using high resolution melt (HRM) analysis has been described for MLVA assays in other organisms (Fortini et al., 2007).

The objective of this study was to evaluate the combined use of MLST and the HRM-MLVA sequence-based methods for discrimination of environmental and clinical *V. parahaemolyticus* isolates previously characterized by other fingerprinting methods, such as PFGE (Ludeke et al., 2014). MLST was selected to identify phylogenetic relationships while MLVA was applied with the hypothesis that it will further discriminate isolates with identical STs. In order to achieve this objective, an HRM-MLVA protocol for rapid and simple characterization of *V. parahaemolyticus* isolates was developed. This is the first study, to the authors' knowledge, to use the combined approach of the subtyping methods MLST, HRM-MLVA, and PFGE for differentiation of *V. parahaemolyticus* isolates.

## Material and methods

### Bacterial strains

For the MLST and MLVA analysis, 58 *V. parahaemolyticus* isolates were selected; among those were 28 environmental (oyster) and 30 clinical isolates. Isolates were selected to represent multiple collection states and serotypes (Table 1). Each isolate was inoculated into Luria Bertani broth with 1% NaCl and incubated with shaking overnight at 35°C. Afterwards, 1 mL of the overnight culture was transferred to a 1.5 mL microcentrifuge tube, heated to 100°C for 10 min, and placed in ice for 5 min. The samples were stored at −20°C until used as a PCR template.

**Table 1:** **Isolates used in this study and their sequence types (ST)**.

**Isolate ID**	**Source of isolate**	**Collection state**	**Serotype**	**tdh**	**trh**	**Allele types**							**ST**
						**dnaE**	**gyrB**	**recA**	**dtdS**	**pntA**	**pyrC**	**tnaA**	
FDA_R2	Oyster	TX	O3:Kut^a^	−	+	86	300	17	55	12	54	86	**729^c^**
FDA_R5	Oyster	TX	O10:Kut	−	+	214	**329**	30	19	**165**	69	26	**730**
FDA_R10	Oyster	FL	O1:Kut	+	+	142	29	10	7	4	24	20	313
FDA_R12	Oyster	LA	O4:K8	+	+	20	25	15	6	7	11	4	32
FDA_R13	Oyster	LA	O4:K10	−	−	**241**	**330**	205	253	28	22	**188**	**732**
FDA_R16	Oyster	FL	O4:K9	+	+	20	25	15	13	7	11	5	34
FDA_R17	Oyster	FL	O4:Kut	−	−	14	30	49	11	49	11	13	536
FDA_R21	Oyster	TX	O5:Kut	−	+	9	21	15	13	4	10	26	12
FDA_R26	Oyster	NJ	O4:K8	+	+	20	25	15	6	7	11	4	32
FDA_R29	Oyster	FL	O11:Kut	−	−	**235**	22	25	273	164	254	20	**734**
FDA_R30	Oyster	FL	O1:Kut	+	+	17	16	UT	36	15	31	26	−
FDA_R45	Oyster	WA	O5:Kut	−	+	37	14	14	9	14	34	26	61
FDA_R47	Oyster	AL	O4:K8	+	+	20	25	15	6	7	11	4	32
FDA_R51	Oyster	AL	O8:Kut	+	+	60	106	31	72	66	62	65	676
FDA_R52	Oyster	WA	O3:Kut	−	+	4	13	11	38	18	46	23	**735**
FDA_R60	Oyster	ME	O10:Kut	−	+	63	**326**	**231**	13	48	120	24	**736**
FDA_R62	Oyster	ME	O1:Kut	−	+	31	327	UT	157	14	3	20	−
FDA_R74	Oyster	VA	O4:K34	−	−	26	58	53	19	28	9	26	108
FDA_R75	Oyster	VA	O8:Kut	+	+	60	106	31	72	66	62	65	676
FDA_R86	Oyster	FL	O6:Kut	−	−	45	**336**	143	7	171	**255**	36	**737**
FDA_R87	Oyster	FL	O8:K70	+	+	145	177	140	158	4	132	104	320
FDA_R94	Oyster	PEI^b^ (Canada)	O3:K5	−	+	47	328	UT	13	2	256	23	−
FDA_R125	Oyster	FL	O11:Kut	+	−	17	**331**	**235**	23	33	137	94	**739**
FDA_R126	Oyster	FL	O4:K42	−	−	36	285	25	250	26	227	26	**740**
FDA_R135	Oyster	SC	O3:Kut	−	−	26	16	41	224	31	32	23	**741**
FDA_R136	Oyster	SC	O1:K20	+	+	31	16	32	36	33	11	19	**775**
FDA_R143	Oyster	FL	O5:Kut	−	−	17	64	137	60	94	11	51	**743**
FDA_R149	Oyster	FL	O1:Kut	+	+	142	29	10	7	4	24	20	313
CDC_K4556_1	Clinical	LA	O1:K25	−	−	31	82	**236**	35	23	26	51	**744**
CDC_K4557	Clinical	LA	O1:K33	−	−	28	4	82	88	63	187	1	**799**
CDC_K4588	Clinical	ME	O5:Kut	−	+	56	16	**237**	8	33	59	20	**746**
CDC_K4857_1	Clinical	HI	O5:K17	−	−	35	43	38	21	31	35	37	79
CDC_K4858	Clinical	HI	O4:K4	−	−	27	84	127	139	54	124	37	283
CDC_K4981	Clinical	OK	O1:Kut	−	−	17	**327**	13	8	172	32	**181**	**748**
CDC_K5009_1	Clinical	MA	O4:K53	+	+	5	71	**238**	162	26	11	107	**749**
CDC_K5010_1	Clinical	MA	O1:Kut	+	−	3	4	19	4	29	4	22	3
CDC_K5058	Clinical	TX	O3:K6	+	−	3	4	19	4	29	4	22	3
CDC_K5067	Clinical	SD	O1:K56	+	+	31	16	13	36	33	11	19	**775**
CDC_K5073	Clinical	MD	O3:K56	+	+	17	57	52	**285**	44	28	36	**750**
CDC_K5125	Clinical	MS	O3:Kut	−	−	195	263	187	75	23	198	**190**	**772**
CDC_K5276	Clinical	NY	O11:Kut	+	+	222	128	21	69	46	236	12	631
CDC_K5278	Clinical	WA	O4:K12	+	+	21	15	1	23	23	21	16	36
CDC_K5282	Clinical	HI	O5:Kut	−	−	19	217	89	175	UT	62	51	−
CDC_K5306	Clinical	GA	O4:K9	+	+	20	25	15	13	7	11	5	34
CDC_K5323_1	Clinical	VA	O5:K17	−	+	83	82	73	83	4	77	58	674
CDC_K5324_1	Clinical	VA	O1:K20	+	+	56	16	32	**286**	14	11	19	**752**
CDC_K5331	Clinical	GA	O4:K8	+	−	11	48	UT	48	26	48	26	−
CDC_K5345_1	Clinical	IA	O4:K12	+	+	21	15	1	23	23	21	16	36
CDC_K5428	Clinical	NV	O1:Kut	+	+	22	28	17	13	8	19	14	199
CDC_K5433	Clinical	WA	O4:Kut	+	+	21	15	1	23	23	21	16	36
CDC_K5436	Clinical	WA	O4:Kut	+	+	21	15	1	23	23	21	16	36
CDC_K5439	Clinical	WA	O4:K8	+	−	11	48	3	48	26	48	26	189
CDC_K5485	Clinical	NC	O6:K18	−	−	29	5	22	12	20	22	25	50
CDC_K5528	Clinical	GA	O4:K68	+	−	3	4	19	4	29	4	22	3
CDC_K5582	Clinical	GA	O11:Kut	+	+	222	128	21	69	46	236	12	631
CDC_K5618	Clinical	NY	O10:Kut	+	+	223	106	31	221	45	171	165	636
CDC_K5621	Clinical	NY	O1:Kut	−	+	39	9	27	39	3	37	30	65
CDC_K5635	Clinical	MD	O5:K30	−	−	158	131	31	**287**	128	43	**189**	**753**

a ut = untypable.

b PEI = Prince Edward Island.

c novel allele type and novel ST in bold.

### Multi-locus sequence typing (MLST)

MLST was performed as described in the protocol for *Vibrio parahaemolyticus* (Gonzalez-Escalona et al., 2008). The PCR products were purified using a PCR purification kit (Qiagen, Valencia, CA) with a total elution volume of 25 μL. The purified samples were sequenced on an ABI 3730 × l sequencer at McLab (South San Francisco, CA). Sequences were analyzed with BioEdit software 7.1.9 (Abbott, Carlsbad, CA). The allelic and sequence type (ST) identification was determined using the MLST database (http://pubmlst.org/vparahaemolyticus). In the cases where whole genome sequence data was available, sequences were *de novo* assembled using CLC Genomics Workbench software 7.0.3 (CLCbio, Germantown, MD) and consensus assemblies submitted to the MLST database for allelic and ST identification.

For identification of clonal complexes, eBURST version 3 was used (http://eburst.mlst.net/). As reported previously, two different STs were considered single-locus variant (SLV) when they differed by a single locus; a double-locus variant (DLV) has two different loci (Gonzalez-Escalona et al., 2008). To be part of a clonal complex, isolates needed to share at least six out of seven alleles.

The minimum evolution tree of the concatenated sequences of the seven loci was built based on the method of Kimura-2-parameter in Mega 6 (Tamura et al., 2013). The ratio between the number of synonymous and non-synonymous substitutions, showing the type of selection at each locus, was calculated using the method of Nei and Gojobori in Mega 6. The hypotheses of neutrality (*d_S_* = *d_N_*), purifying selection (*d_S_*/*d_N_* > 1), and positive selection (*d_S_*/*d_N_* < 1) were tested.

### Multiple-locus variable-number tandem-repeat analysis (MLVA)

Three multiplex real-time-PCR assays using HRM curve analysis were performed with the LightCycler® 480 High Resolution Melting Master Kit (Roche, Indianapolis, IN). The primer sequences utilized in this study are listed in Table 2 and have been previously described (Harth-Chu et al., 2009). These primers were utilized as unmodified oligonucleotides since HRM was used as the detection method rather than CE. Each reaction mixture for the multiplex PCR A and B (Multi A and B) had a final volume of 20 μL and consisted of: 1X master mix solution (Roche), 2 mM MgCl_2_ (Roche), 0.2 μM each primer (Integrated DNA Technologies; IDT, Coralville, IA), and 5 μL boiled template. For multiplex PCR C (Multi C), reaction concentrations were the same as for Multi A and B, except for primer pair VP2-07; 0.4 μM of each primer was used. The temperature program was as described previously for Multi A and B (Harth-Chu et al., 2009): Initial denaturation at 95°C for 15 min, followed by 20 cycles of a touchdown PCR consisting of denaturation at 94°C for 30 s, annealing starting at 63°C and decreasing 0.2°C per cycle for 1.5 min, and elongation at 72°C for 1 min. A final 10 cycles of denaturation at 94°C for 30 s, annealing at 59°C for 1.5 min, and elongation at 72°C for 1 min was used. The cycling program of Multi C included the same initial denaturation as for Multi A and B; cycling consisted of 30 cycles of denaturation at 94°C for 30 s, annealing at 61°C for 1.5 min, and elongation at 72°C for 1 min. A final annealing step at 60°C for 30 min completed both cycling programs. The melt curve analysis was performed using the Roche High Resolution Melting Kit protocol. The temperature program for the HRM analysis started at 95°C for 1 min with a ramp of 4.4°C per s, followed by 40°C for 1 min with a ramp of 2.2°C per s, 60°C for 1 s with a ramp of 4.4°C per s and a continuous step at 95°C. For the PCR amplification and HRM analysis a LightCycler® 480 (Roche) was used.

**Table 2:** **Primer sequences including their amplification product length and melting temperature for MLVA multiplex PCR**.

**Multiplex-PCR**	**Locus**	**Primer**	**5′-3′ Sequence**	**Product length [bp]**	**Melting temperature Tm [°C]**	**References**
Multi A	VP1-11	VP1-11 F	CTGCCTGGAGAATTGGCTTA	854	95	8
		VP1-11 R	TGAGCCTGAAGCTGAAAACA			
	VP2-03	VP2-03 F	CATAAACGAGCGACACGAGA	168	57	8
		VP2-03 R	GCGCAAAAATTCATTGTGATT			
	VPTR5	VPTR5 F	GCTGGATTGCTGCGAGTAAGA	204	82	22
		VPTR5 R	AACTCAAGGGCTGCTTCGG			
	VPTR7	VPTR7-1F	TATCTACAAAGGTGGCGGAGAT	200	80	8
		VPTR7-1R	AAGGTGTTACTTGTTCCAGACG			
Multi B	VP1-17	VP1-17 F	TCAACACGAGCTTGATCACC	206	69	8
		VP1-17 R	GAAATCCGGAGTACCTGCAA			
	VP1-10	VP1-10 F	CGTCTTGCTCGTGAACGTAA	955	94	8
		VP1-10 R	TCATTAAGTCAGGCGTGCTG			
	VPTR1	VPTR1 F	TAACAACGCAAGCTTGCAACG	253	54	22
		VPTR1 R	TCATTCTCGCCACATAACTCAGC			
	VPTR8	VPTR8 F	ACATCGGCAATGAGCAGTTG	301	89	22
		VPTR8 R	AAGAGGTTGCTGAGCAAGCG			
Multi C	VP2-07	VP2-07 F	TGATTTTGAAGCAGCGAAGA	296	98, smaller peak at 74	8
		VP2-07 R	TTTGTGACTGCTGTCCTTGC			
	VPTR3	VPTR3 F	CGCCAGTAATTCGACTCATGC	331	77	22
		VPTR3 R	AAGACTGTTCCCGTCGCTGA			
	VPTR4	VPTR4 F	AAACGTCTCGACATCTGGATCA	227	85	22
		VPTR4 R	TGTTTGGCTATGTAACCGCTCA			
	VPTR6	VPTR6 F	TGTCGATGGTGTTCTGTTCCA	316	107, smaller peak 97, 72	22
		VPTR6 R	CTTGACTTGCTCGCTCAGGAG			

Due to overlapping peaks in Multi A and C, the presence of the target loci VNTR5 and VNTR7, VNTR3, and VP2-07 were confirmed using simplex real-time-PCR for each gene with the same PCR reaction conditions and cycling parameters as described for the multiplex, but with omission of the three other primer sets. The melt curves were analyzed with BioNumerics software 6.6 with a customized script (Applied Maths, Austin, TX). This script compares the melting curves of each multiplex PCR, as well as a combination of all curves from the three multiplex PCRs. The combined dendrogram of all three multiplex PCR was built based on the Pearson correlation of average trend curves in BioNumerics as well. This dendrogram was also converted to a rendered rooted tree.

## Results

### Multi-locus sequence typing (MLST)

From the 58 *V. parahaemolyticus* isolates analyzed, MLST resulted in 41 different STs (Table 1). Four (6.9%) and one (1.7%) of the isolates were untypeable for *recA* and *pntA*, respectively. For those strains, no ST could be assigned. Twelve (42.9%) of the oyster and ten (40.0%) of the clinical isolates were a novel ST. The most frequently identified STs were ST36 (13.3%) and ST3 (10.0%) in clinical isolates and ST32 (10.7%), ST313 (7.1%), and ST676 (7.1%) in oyster isolates.

All loci showed ratios of synonymous and non-synonymous substitutions (*d_N_*/*d_S_*) below 1 and therefore under purifying selection, as expected for housekeeping genes. eBURST analysis divided the 53 isolates for which a ST could be identified into 37 singletons and two groups: one SLV and one DLV (data not shown). No clonal complexes could be identified; demonstrating that none of the STs identified in this study share more than six alleles and, therefore, belong to different *V. parahaemolyticus* lineages.

**Figure 1:**
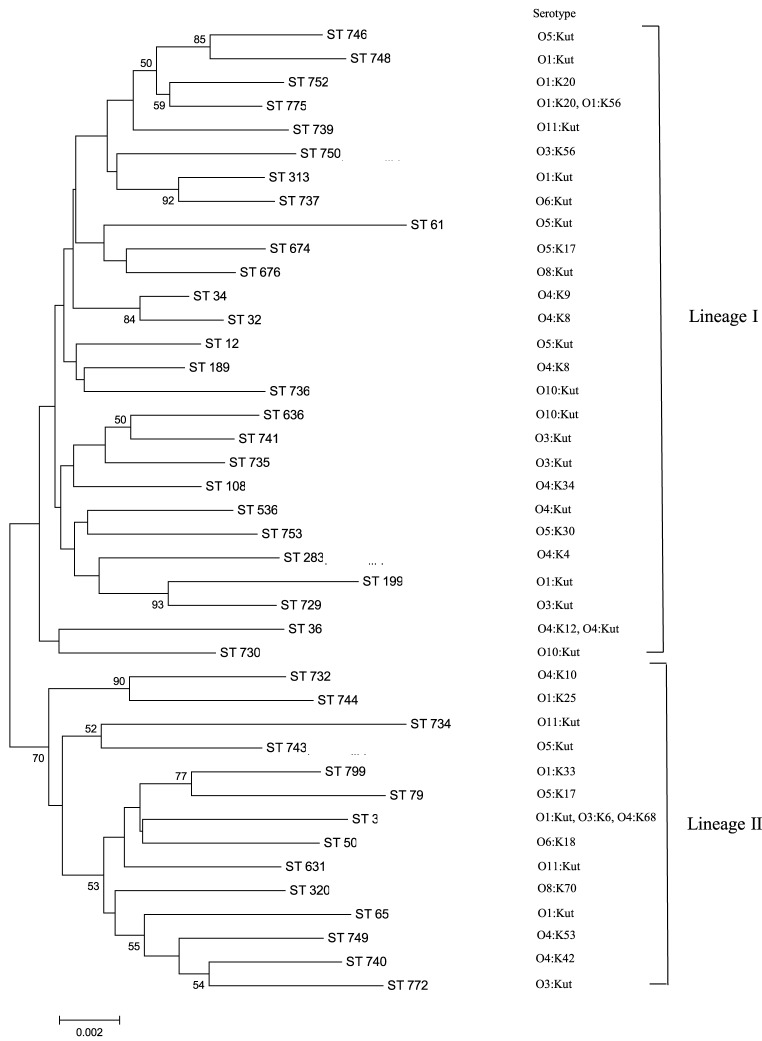
**MLST minimum evolution tree of the 58** ***V. parahaemolyticus*** **isolates**. The tree was built with Mega six software using concatenated sequences. The scale represents the evolutionary distance and the branches show bootstrap values above 50%.

A minimum evolution tree was constructed using the concatenated sequences of each allele (Figure 1). The isolates grouped into two main clusters, or lineages (I and II), with each lineage containing ST of clinical and oyster isolates. Isolates with the same ST generally had the same serotype; ST631 isolates possessed serotype O11:Kut, ST676 were serotype O8:Kut, ST36 were serotype O4:K12 or O4:Kut, and ST313 were serotype O1:Kut. However, the three ST3 isolates had all different serotypes.

### Multiple-locus variable-number tandem-repeat analysis (MLVA)

The 58 *V. parahaemolyticus* isolates from this study were further analyzed by MLVA with HRM analysis. Three multiplex PCRs covering twelve different loci were used. Each multiplex PCR generated reproducible melting curve profiles of select isolates (data not shown). Table 3 shows the percentage of isolates from which each target sequence in the MLVA scheme was amplified. From the Multi A, VP2-03, VPTR7, or VP1-11 was not amplified in 10% and 14.3%, 46.6% and 39.3%, or 3.3% and 3.6% of clinical and oyster isolates, respectively; VPTR5 was amplified from all isolates. In Multi B, VP1-10 or VPTR8 was not present in 96.7% and 17.9%, 23.3% and 17.9% of clinical and oyster isolates, respectively; VPTR1 and VP1-17 was amplified in all isolates with the exception of 6.7% of clinical isolates not amplifying VPTR1. From Multi C, VPRT6 was not amplified in 83.3% and 85.7% of clinical and oyster isolates, respectively. All isolates amplified VPTR4, VPTR3, and VP2-07, with the exception of 3.6% of oyster isolates for VPTR4.

**Table 3:** **Presence of individual MLVA genes in clinical and oyster isolates**.

	**Multi A**				**Multi B**				**Multi C**			
	**VP2-03**	**VPTR7**	**VP1-11**	**VPTR5**	**VP1-10**	**VPTR1**	**VPTR8**	**VP1-17**	**VPTR4**	**VPTR3**	**VPTR6**	**VP2-07**
Clinical isolates (*n* = 30)	27	16	29	30	1	28	23	30	30	30	5	30
*Percentage (%)*	*90.0*	*53.3*	*96.7*	*100.0*	*3.3*	*93.3*	*76.7*	*100.0*	*100.0*	*100.0*	*16.7*	*100.0*
Oyster isolates (*n* = 28)	24	17	27	28	23	28	23	28	27	28	4	28
*Percentage (%)*	*85.7*	*60.7*	*96.4*	*100.0*	*82.1*	*100.0*	*82.1*	*100.0*	*96.4*	*100.0*	*14.3*	*100.0*

Using the trend curves of each individual multiplex PCR, dendrograms were constructed (data not shown). Each multiplex PCR generally clustered the isolates based on their serotypes and ST. The individual multiplex PCR dendrograms demonstrated the ability of MLVA to differentiate between the same ST (data not shown).

### Comparison of MLST, MLVA, and PFGE

**Figure 2:**
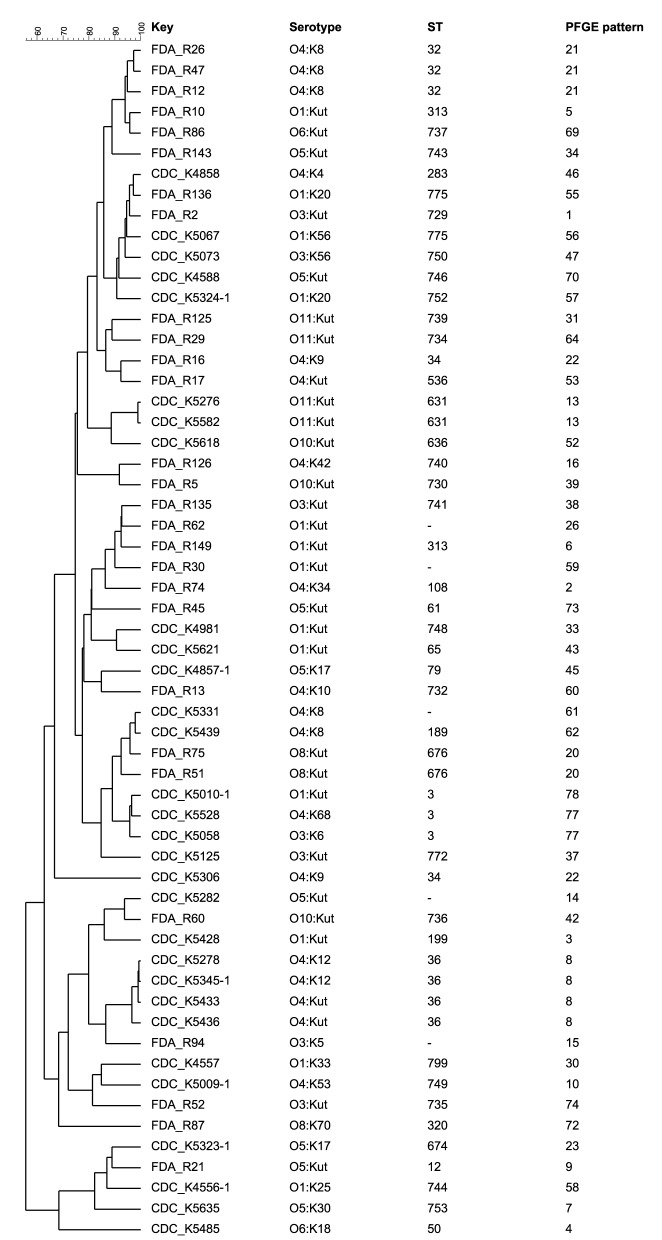
**Combined dendrogram of MLVA melting curves of the three multiplex PCRs built with BioNumerics software version 6.6. using Pearson correlation and the unweighted pair group method using arithmetic averages (UPGMA)**. Isolates originated from oysters starting with “FDA,” isolates from clinical origin labeled with “CDC.” The PFGE pattern designations are as previously reported (Ludeke et al., 2014).

**Figure 3:**
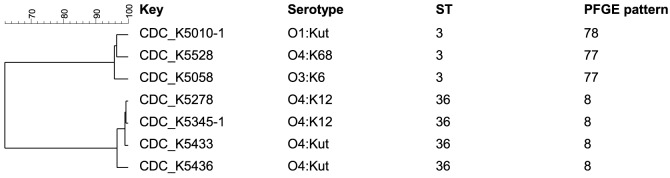
**Dendrogram of MLVA melting curves of the three multiplex PCRs for the isolates carrying ST3 and ST36 built with BioNumerics software version 6.6. using Pearson correlation and the unweighted pair group method using arithmetic averages (UPGMA)**. The PFGE cluster designations are as previously reported (Ludeke et al., 2014).

Based on the hypothesis MLVA can differentiate isolates with the same ST and PFGE pattern, these isolates' MLVA patterns were compared to the MLST data, as well as previously published PFGE results (Ludeke et al., 2014). To compare these methods, dendrograms were built of the combined melting curves from the three MLVA multiplex PCRs and correlated to the PFGE cluster and ST of each isolate. MLVA allowed further differentiation of isolates with identical STs and PFGE clusters (Figure 2). Specifically, the isolates with ST3 and ST36 share the same PFGE cluster, but were distinguishable by MLVA melting curve profiles (Figure 3). The dendrogram with only ST3 and ST36 isolates showed ST-specific clusters, but separation within those clusters based on the combined melting curves of MLVA.

## Discussion

This study analyzed 58 *V. parahaemolyticus* isolates by MLST and a newly-developed HRM-MLVA assay to investigate the relatedness of the isolates. The seven gene MLST protocol reported in a previous study was employed in this study (Gonzalez-Escalona et al., 2008). A different MLST method using ten housekeeping genes was described (Yan et al., 2011). Both methods report the same level of discrimination; however, the seven gene protocol was selected due to the availability of a public repository for the data (http://pubmlst.org/vparahaemolyticus), which allows comparisons to be made with isolates analyzed by other researchers. With 22 novel STs, this study substantially contributed to the diversity in the MLST database. Four of our isolates were untypeable for *recA.* In a recently published study, a *V. parahaemolyticus* strain contained a *recA* gene that was fragmented by a 30 kb DNA insertion (Gonzalez-Escalona et al., 2015). It is possible a similar insertion exists in the *recA* gene of some of the strains in the current study, but further analysis is needed to confirm.

ST3, ST32, and ST36 were the STs that occurred most often in our isolates, as well as in the public database. In our study, the fourth most frequent ST observed was ST676, which is one of the novel STs reported here. Two of these STs (ST3 and ST36) have been reported as part of clonal complexes CC3 and CC36, respectively (Gonzalez-Escalona et al., 2008), and correlated with outbreaks in multiple countries, including the US and Chile (Fuenzalida et al., 2006; Martinez-Urtaza et al., 2013). ST3 was identified as the ancestral ST of CC3 with ST27, ST42, and ST51 as SLVs. None of other STs from CC3 were identified in this study. A previous study using MLST on a set of clinical and environmental isolates from the Pacific Northwest Region of the United States showed that some environmental isolates were of ST3, suggesting a higher potential for virulence than other environmental isolates (Turner et al., 2013). In this study, three clinical isolates were ST3 and no direct relationship to environmental or other clinical clades were observed.

We developed an HRM-MLVA method, based on an existing MLVA method that uses CE, for subtyping of *V. parahaemolyticus* and to differentiate between similar PFGE patterns or STs. The CE method provides the actual number of tandem repeats while HRM does not. However, the HRM analysis still recognizes allelic variants and is able to distinguish between otherwise indistinguishable strains. For example, the ST3, ST32, and ST36 strains in this study also shared common PFGE profiles, but each isolate produced a unique HRM curve combination. This resolving power of HRM-MLVA is similar to previous reports of CE-MLVA, where Chilean isolates which shared a DGREA pattern and were ST3 could be differentiated by CE-MLVA (Gonzalez-Escalona et al., 2008; Harth-Chu et al., 2009). These data demonstrate that the HRM-MLVA method developed in this study provides similar discrimination as previously reported for the CE-MLVA method and is suitable examination of *V. parahaemolyticus* isolates. Additionally, the use of HRM-MLVA on the LightCycler®480 saves the step of electrophoretic detection, thus minimizing the potential for cross contamination by PCR amplicons during that additional handling step.

The loci amplified in this study by MLVA are coding proteins such as a putative hemolysin (VPTR4) and putative collagenase (VPTR3) (Kimura et al., 2008). Most of these genes, could be amplified from the current strain selection with the exception of VPTR7 (Multi A), VP1-10 (Multi B), and VPTR6 (Multi C). Nearly all clinical isolates failed to amplify the VP1-10 locus and approximately half failed to amplify the locus VPTR7. A failure to amplify VPTR7 from some shellfish isolates has been reported previously (Harth-Chu et al., 2009). Fewer than 20% of isolates amplified VPTR6 in this study. Additionally, a previous study found VPTR6 to be one of the few loci with high genetic diversity (Harth-Chu et al., 2009). Together, these data suggest that these two loci might not be suitable targets for future MLVA studies, especially for environmental isolate screening. Nonetheless, the HRM-MLVA method successfully discriminated between otherwise indistinguishable *V. parahaemolyticus* isolates.

Previous studies have employed multiple methods for characterization and subtyping of *V. parahaemolyticus* isolates from various sources. For example, DePaola et al. used a combination of serotyping and ribotyping to identify types more highly associated with clinical isolates (DePaola et al., 2003). Turner et al. utilized REP-PCR, as fingerprint-based subtyping method, followed by MLST to identify region-specific clades of *V. parahaemolyticus* (Turner et al., 2013). Banerjee et al. employed PFGE, MLST, serotyping, and ribotyping to examine clinical *V. parahaemolyticus* isolates and provided combinatorial analysis to determine relatedness (Banerjee et al., 2014). However, none of these previous studies utilized the combination of two highly discriminatory, sequence-based methods as does the current study.

This combined method approach described here using PFGE, MLST, and MLVA has not been previously reported for discrimination of *V. parahaemolyticus* isolates, but is similar to approaches used for other organisms: for example, the Centers for Disease Control and Prevention has used a combination of PFGE followed by MLVA for epidemiological investigations of STEC O157 to discriminate between closely related isolates (Hyytia-Trees et al., 2006). Also, a combination of MLST and MLVA has been used as an epidemiological tool for distinguishing between clones of *Listeria monocytogenes* (Chenal-Francisque et al., 2013).

This study used a combined method approach to increase the discrimination of *V. parahaemolyticus* isolates. MLST was able to determine the identity and the phylogenetic relatedness of the isolates in this collection. As hypothesized, the developed HRM-MLVA method further refined the relationship of isolates by being able to distinguish between isolates with indistinguishable PFGE groupings or STs. Our data demonstrates that a combination of PFGE, MLST, and HRM-MLVA would be the most suitable approach for outbreak and evolutionary investigations of *V. parahaemolyticus*, due to the high resolution provided. In instances where further discrimination is needed, and if available, next generation sequence data could be used to determine relatedness or to generate subtyping results *in silico*.

### Conflict of interest statement

The authors declare that the research was conducted in the absence of any commercial or financial relationships that could be construed as a potential conflict of interest.
